# Cytological Diagnosis Suggesting Candidal Infection of the Nasolacrimal Duct in an Uncontrolled Diabetic Patient With Gingival Abscess

**DOI:** 10.7759/cureus.44257

**Published:** 2023-08-28

**Authors:** Veda Samhitha NS, Priyathersini N, Barathi Gunabooshanam, Ponneyinchelvi AS, Prasanna Kumar S

**Affiliations:** 1 Department of Pathology, Sri Ramachandra Institute of Higher Education and Research, Chennai, IND; 2 Department of Otorhinolaryngology, Sri Ramachandra Institute of Higher Education and Research, Chennai, IND

**Keywords:** nasolacrimal duct obstruction, fine needle aspiration cytology (fnac), dacryostenosis, sando, pando, fungal organisms, budding yeast, candida, cytology, nasolacrimal duct

## Abstract

In this unique cytology case of a 64-year-old diabetic male who presented with left-sided facial swelling between the ala and lateral canthus of the left eye, conventional fine-needle aspiration cytology (FNAC) was done.

FNAC of the swelling showed desquamated epithelial cells from the nasolacrimal duct and abundant proteinaceous material admixed with fungal organisms like that of budding yeast forms, morphologically resembling Candida on May-Grunwald-Giemsa stain. Special stain with periodic acid-Schiff revealed positivity for budding yeast forms.

In this case report, we discuss the causes and clinical effects of nasolacrimal duct obstruction, cytological diagnostic features, and microscopic recognition of fungal organisms on routine staining as well as on special fungal stains.

## Introduction

Nasolacrimal duct obstruction (NLDO) or dacryostenosis is the narrowing of the drainage pathway of tears due to a congenital or acquired etiology.

Acquired nasolacrimal duct obstruction (ANDO) is subclassified into idiopathic primary acquired nasolacrimal duct obstruction (PANDO) [1] and secondary acquired nasolacrimal duct obstruction (SANDO). The causes [2] associated with SANDO may vary from infections, inflammation, mechanical obstruction, trauma, or neoplastic lesions.

Here we present a case of infection leading to SANDO.

## Case presentation

A 64-year-old male came with the chief complaint of left-sided facial swelling, associated with pain for one month. The patient reported that there has been an increase in the size of the swelling since then. He was not aware if he had any comorbidities and neither did he undergo any prior treatment for the current illness.

**Figure 1:**
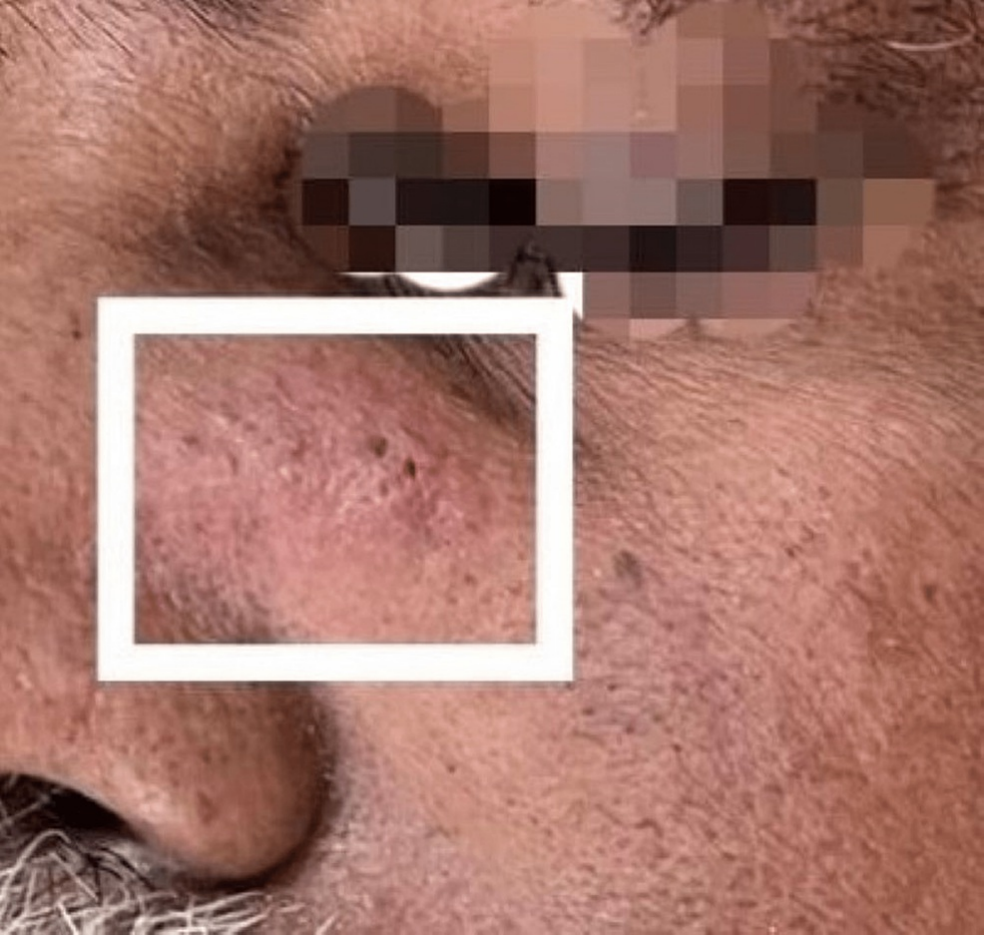
A swelling of size 4 x 3 cm noted above the tip of ala (white square)

On local examination, a swelling of size 4 x 3 cm (Figure 1) was located just adjacent to the left lateral wall of the nose, 1 cm above the tip of ala. The skin over the swelling appeared erythematous. Warmth and tenderness were felt over the swelling.

During the ophthalmological examination, an erythematous left punctum with conjunctival injection was noted with expressible watery punctual discharge.

Systemic examination was unremarkable except for an incidental detection of a 0.5 x 0.5 cm bleb over the left upper canine gingiva with dental caries.

The patient was subjected to routine blood and urine investigations.

There was a significant elevation in blood glucose levels, both fasting and postprandial. Hemoglobin A1C (HbA1C) was on the higher side of 12.6%.

Urine for ketones was negative. Diagnostic nasal endoscopy showed a deviated nasal septum to the right. Contrast-enhanced computerized tomography (CECT) of the paranasal sinuses showed normal paranasal sinuses. An incidental detection of a peripherally enhancing soft tissue hypodense lesion of the right buccal mucosa without any underlying bone erosion, favoring an infective etiology, was made and a suggestion to rule out a neoplastic etiology was given. Pus culture and sensitivity sample sent from left gingival abscess detected Streptococcal species.

**Figure 2:**
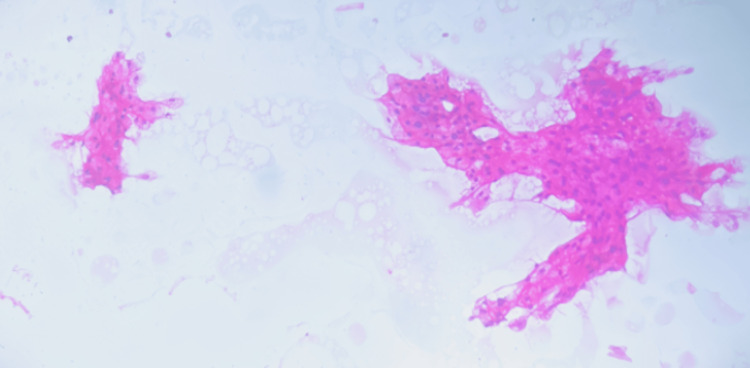
Clusters of mature squamous epithelial cells from nasolacrimal duct with bland nuclear features (fine-needle aspiration cytology, H&E stain 10x magnification)

Conventional fine-needle aspiration cytology (FNAC) of the facial swelling was done, which showed clusters of mature squamous epithelial cells from the nasolacrimal duct with bland nuclear features (Figure 2) admixed with scattered inflammatory cells composed of neutrophils and a few lymphocytes in a proteinaceous background. 

**Figure 3:**
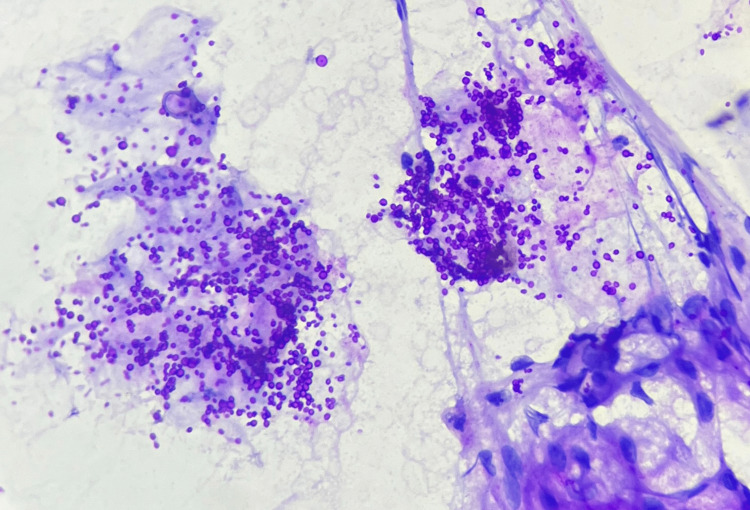
Fungal budding yeast forms that morphologically resemble Candida (fine-needle aspiration cytology, May-Grunwald-Giemsa stain, 40x magnification)

**Figure 4:**
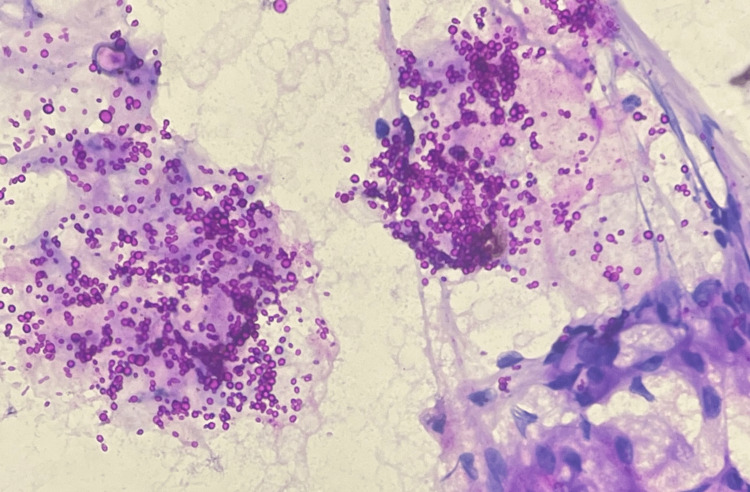
Budding fungal yeast forms resembling Candida (fine-needle aspiration cytology, periodic acid-Schiff stain, 40x magnification)

**Figure 5:**
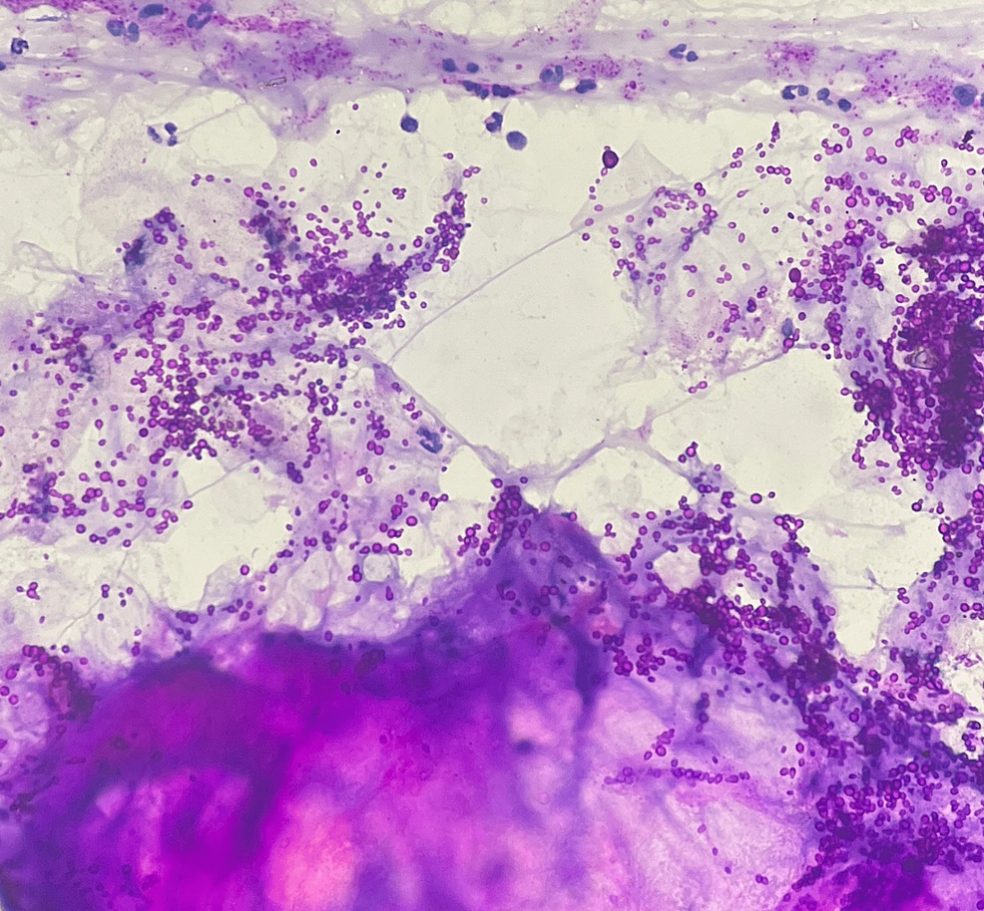
Fungal yeast forms admixed with abundant proteinaceous secretions from the nasolacrimal duct (fine-needle aspiration cytology, periodic acid-Schiff stain, 40x magnification)

FNAC from the facial swelling was reported to have an ongoing inflammation and abundant proteinaceous secretions admixed with fungal organisms suggestive of budding yeast forms of Candida which were seen on May-Grunwald-Giemsa stain and highlighted by periodic acid-Schiff stain (Figures 3-5). No malignant cells were seen.

The patient received liberal saline washes to provide symptomatic relief of the NLDO. The patient received intravenous antibiotics and injectable hypoglycemics. Advice for maintenance of better oral hygiene practices and planning for dental extraction on a later date after control of infection with drainage of the gingival abscess were done.

## Discussion

According to a retrospective study performed in a tertiary care hospital [3] in India for five years, 20,102 patients were found to have lacrimal drainage disorders. The average age of NLDO was 68.3 ± 14.9 years and 34.4% were males.

PANDO accounted for 51.56%, congenital nasolacrimal duct obstruction (CNLDO) accounted for 26.8% followed by SANDO which accounted for 21.7%.

More than two-thirds of the patients presented with epiphora while one-third came with facial swelling. Culture positivity of infectious causatives was established in 51.9% of cases, among which 96.5% were bacterial and 3.5% were fungal. Candida comprised 2% of all infections. SANDO [4] is seen to occur in association with infections, inflammatory causes, trauma, mechanical causes, or neoplastic lesions. Goblet cell dysfunction along the ductal lining along with fibrosis, luminal narrowing, desquamated cells, and accumulation of thick secretions create a favorable environment for the growth of microbial organisms [4]. The source of infection can be conjunctiva, nasal cavity, or paranasal sinuses in individuals with risk factors such as poor hygiene, narrow nasal bony canal, excessive or reflux lacrimation, deviated nasal septum, or hypertrophied inferior turbinate. Common organisms found are *Staphylococcus* and *Streptococcus* species [5], while fungi are rarely encountered.

Periodic acid-Schiff and Grocott methenamine silver stains highlight the contour of the fungal forms. Candida are yeast forms that reproduce by budding and form pseudohyphae that need to be differentiated from true hyphae. Candidal pseudohyphae appear elongated without septations and are more slender when compared to true hyphae.

## Conclusions

A state of immunosuppression due to hyperglycemia has put this patient at a higher risk of developing infections. The end results of dacryostenosis vary from secondary infections, mucocele, pyocele, chronic conjunctivitis, orbital cellulitis, and orbital abscesses. Recognition of the cause is important to treat and prevent progression to the above-mentioned complications.
